# Stretching Out: Leopard Home‐Range Expansion in Response to Suppressed Population Density in a Recovering Post‐War Landscape

**DOI:** 10.1002/ece3.72312

**Published:** 2025-10-10

**Authors:** Willem D. Briers‐Louw, Tamar Kendon, Andres Hayes, David Gaynor, Vincent Naude

**Affiliations:** ^1^ Department of Conservation Ecology and Entomology Stellenbosch University Matieland South Africa; ^2^ Zambeze Delta Conservation Foundation Marromeu Sofala Mozambique; ^3^ Mammal Research Institute University of Pretoria Pretoria South Africa

**Keywords:** AKDE, collaring, large carnivore, *Panthera pardus*, population density, spatial ecology

## Abstract

Large carnivores have profound regulatory effects on ecosystems and provide substantial socio‐economic benefits. However, mounting anthropogenic pressures are driving their global decline, threatening many species with extinction. Leopards, in particular, face challenges due to their wide‐ranging behavior, which exposes them to conflict with people and bycatch snaring, highlighting the importance of understanding their spatial ecology to develop more effective conservation strategies. This study aimed to estimate the home‐range size of female leopards (*n* = 4) within the Zambezi Delta, a unique mesic landscape undergoing broad‐scale ecological recovery in central Mozambique. Home‐range sizes ranged from 46 to 365 km^2^, falling within the range of global estimates. Surprisingly, these home‐range sizes, along with additional parameter estimates such as daily distance moved and home‐range overlap, were most comparable with those reported in arid environments. With female leopards appearing to occupy larger areas than theoretically required based on energetic needs, it is plausible that their spatial ecology is likely influenced by low population density. As the population recovers, we anticipate a re‐structuring of socio‐spatial dynamics driven by dispersal‐regulated processes, with females likely contracting their home‐range and partitioning them to their philopatric daughters. This study provides the first robust estimation of leopard home ranges in Mozambique and provides critical insights into the spatial ecology of leopards in a post‐war landscape. We recommend long‐term monitoring to track changes in population demographics and socio‐spatial dynamics as restoration efforts continue across central Mozambique.

## Graphical abstract

Leopards are ecologically, economically, and culturally significant, yet they face growing threats from anthropogenic activities which impact their population demographics and spatial ecology. This study estimated the home‐range size of female leopards in the mesic Zambezi Delta landscape of central Mozambique, revealing ranges between 46 and 365 km^2^, which are more comparable to those observed in arid environments, possibly due to low population density. These findings offer insights into leopard spatial ecology in a post‐war landscape and highlight the need for long‐term monitoring to track demographic and socio‐spatial changes as this ecosystem undergoes recovery.

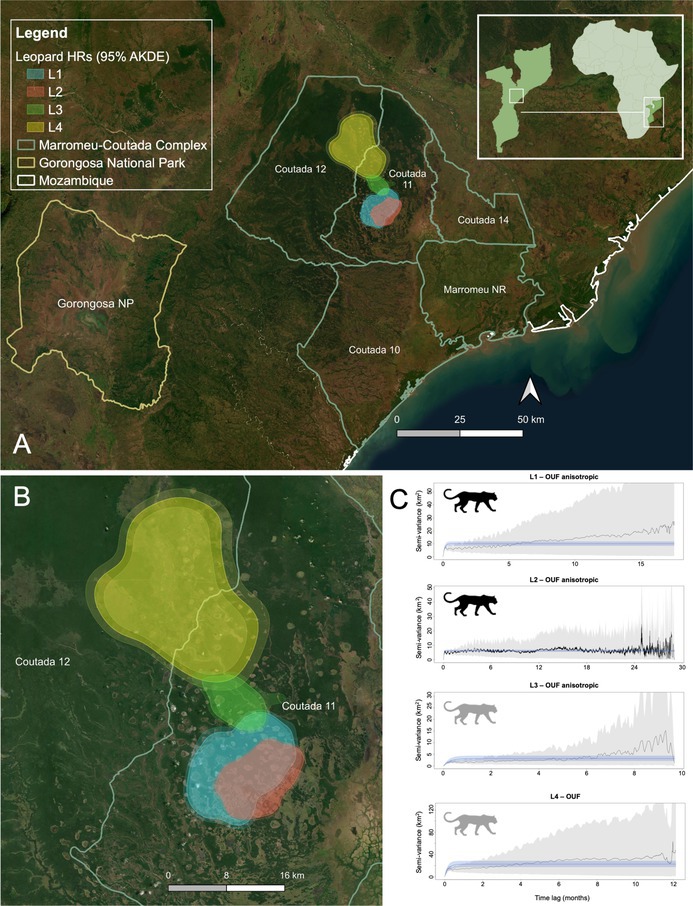

## 1 Introduction

Large carnivores drive complex ecological processes through their regulatory effect on trophic levels (e.g., herbivore and mesopredator population regulation), which helps structure ecosystems and restore resilience (Estes et al. 2011). Their value also extends along the economic axis by contributing substantially to ecotourism and sustainable wildlife economies (Lindsey et al. 2007). Yet, despite their importance, mounting anthropogenic pressures are driving large carnivore declines globally, where many species are now threatened with extinction (Estes et al. 2011; Ripple et al. 2014). Given their wide‐ranging spatial behavior and the physical threat posed to people and livestock, large carnivores are particularly prone to conflict with humans (Ripple et al. 2014). Consequently, improving our understanding of their spatial ecology is imperative for the effective conservation of these ecologically, economically, and culturally significant species, especially in landscapes significantly transformed by anthropogenic activities.

Leopards ( *Panthera pardus* ) are solitary, elusive large carnivores with cryptic behaviors that are challenging to study. Leopards require extensive home ranges (HRs) to satisfy their considerable energetic demands (Weise et al. 2015), which vary substantially across their geographic range, from 5.65 km^2^ in high‐density, mesic habitats (Le Roux et al. 2022) to over 2000 km^2^ in low‐density, arid regions (Bothma et al. 1997). While conspecific density and prey biomass are strong drivers of leopard HR size (Le Roux et al. 2022; Marker and Dickman 2005), their spatial distribution may also be influenced by interspecific interactions with other large predators, such as lions ( *Panthera leo* ). However, such competitive effects appear negligible at low predator densities (Loveridge et al. 2022), and leopards may exhibit minimal behavioral modifications towards dominant competitors, even at high densities (Balme et al. 2017). Despite their remarkable adaptability, particularly within anthropogenically altered landscapes, leopards are listed as Vulnerable due to ongoing population and range declines (IUCN Red List; Stein et al. 2023). Contemporary research identifies a positive correlation between leopard HR size and human population density (Snider et al. 2021); this relationship is likely driven by elevated anthropogenic pressures (e.g., direct mortality, prey base depletion, and habitat loss) which cause declines in leopard density and increases in leopard HR size (Fattebert et al. 2015). Larger HRs may expose leopards to greater risks, particularly at the peripheries of protected areas, where individuals are more vulnerable to detrimental edge effects (Woodroffe and Ginsberg 1998). These effects may suppress population growth and hinder recovery, thereby underscoring the complex challenges that characterize leopard conservation efforts.

In post‐conflict landscapes, large carnivores often occur at diminished densities due to prolonged anthropogenic pressures (Braga‐Pereira et al. 2020; Briers‐Louw, Kendon, Rogan, et al. 2024; Gaynor et al. 2021). Such demographic disturbances can exert enduring impacts on leopard socio‐spatial dynamics, potentially altering movement patterns and spatial utilization (Fattebert et al. 2016). Where populations remain unstable or occur below ecological carrying capacity, resource acquisition may become the primary driver of HR size, while in more extreme instances, natural processes such as dispersal patterns may be disrupted by unsustainable levels of anthropogenic pressure (Fattebert et al. 2015; Naude et al. 2020). Conversely, when populations receive adequate protection and are released from such anthropogenic pressure, female leopards typically exhibit HR contraction as densities increase, and form matrilineal kin clusters, with mothers often relinquishing portions of their HR to their philopatric daughters (Fattebert et al. 2016). Such patterns of recovery have been documented in non‐conflict settings in South Africa, such as the re‐colonization of leopards in the Western Cape following a shift in land‐use practice (McManus et al. 2024) and leopard population recovery following increased regulation of problem animal control and trophy hunting in KwaZulu‐Natal (Balme et al. 2009). While these drivers differ from post‐conflict recovery, both cases emphasize the value of reducing anthropogenic‐related mortality in promoting the restoration of large carnivore populations across their range. Given the challenges of monitoring leopards, relatively few comparative landscape studies exist and contribute to our understanding of their spatial ecology in the context of anthropogenic disturbance and potential for population recovery.

Effectively guiding conservation strategies of threatened species requires baseline assessments of population status and information on spatio‐dynamic responses to landscape change. Decades of war led to significant wildlife declines, especially in central Mozambique, where these impacts were most severe (Hatton et al. 2001). While leopards have persisted in this region, their densities remain low and seemingly suppressed by the long‐term impacts of warfare (Briers‐Louw, Kendon, Rogan, et al. 2024). There remains a notable paucity of data on leopard spatial ecology, both in Mozambique and other post‐conflict landscapes. The aim of this study was to estimate HR size and associated spatial parameters of female leopards in the Zambezi Delta of central Mozambique. We contextualize these findings relative to range‐wide leopard HR estimates, discussing potential factors influencing such spatial ecology and the consequences thereof in a recovering post‐war landscape.

## 2 Methods

### 2.1 Study Area

**FIGURE 1:**
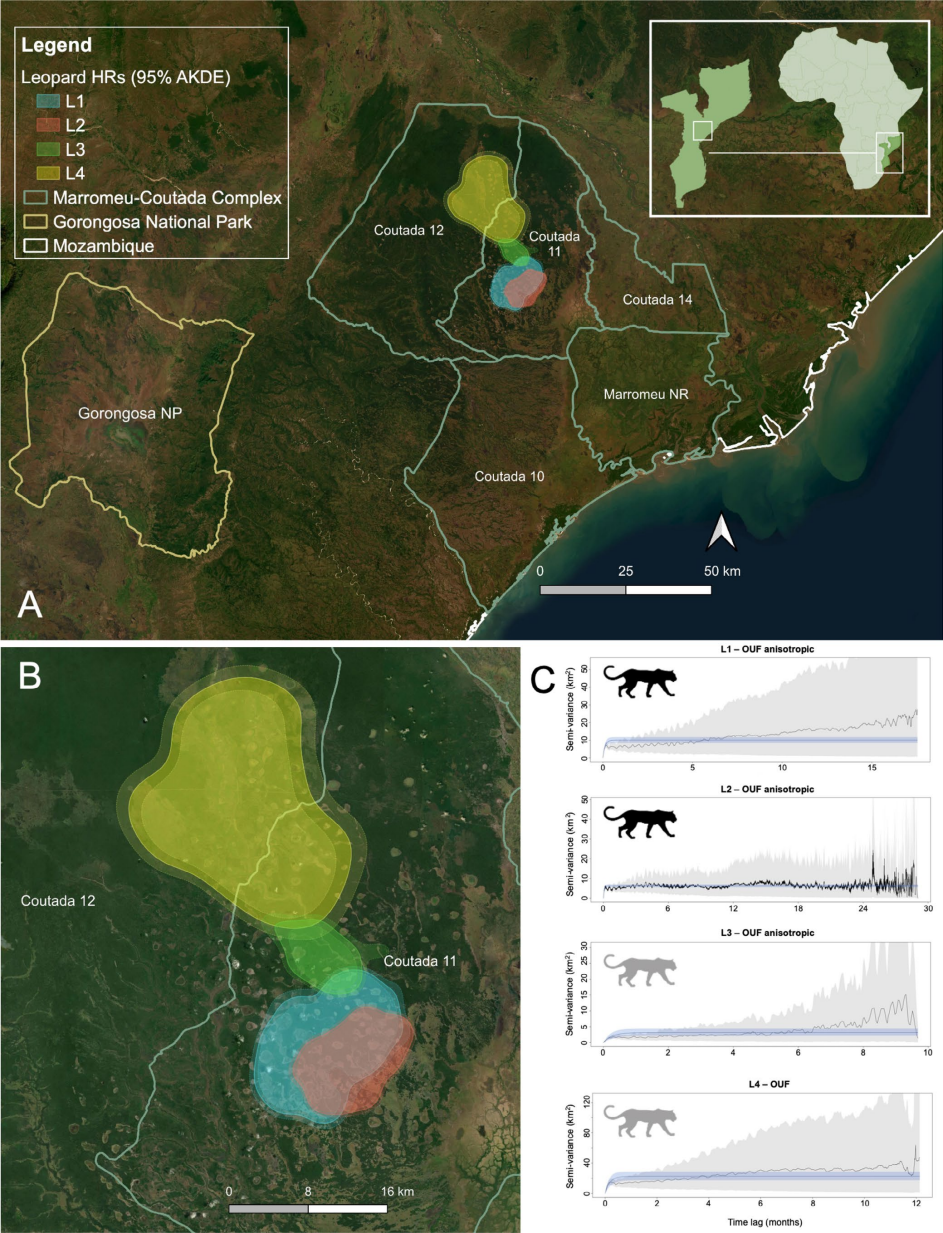
Leopard home‐range (HR) estimations were implemented in the Zambezi Delta in central Mozambique between 2019 and 2023 (A). HR estimates based on autocorrelated kernel density estimations (AKDEs) at the 95% isopleth are indicated by solid polygons, with lower and upper 95% confidence intervals (CIs) represented by dotted lines and a lighter shade (B). Semivariance plots indicate range stabilization for each leopard, with the leopard ID and best‐fitting models displayed above each plot (C). Older, more mature females (> 4 years old) are represented by black leopard icons, while younger, less mature females (< 4 years old) are represented by gray leopard icons.

The Marromeu Complex covers 9750 km^2^ in the southern half of the Zambezi Delta in central Mozambique (Figure 1). This landscape is globally recognized as a RAMSAR Wetland Site owing to its considerable size and immense value for biodiversity, ecosystem services, and human livelihoods (Beilfuss et al. 2010). The Complex consists of the Marromeu National Reserve and four wildlife management areas (WMAs), namely Coutadas 10, 11, 12, and 14, each independently protected and managed, but with increasing collaboration and joint patrols between neighboring areas. The climate is tropical with defined dry (June–November) and wet seasons (December–May), and a mean annual rainfall of 1000–1400 mm. This low‐lying landscape comprises a forest‐floodplain mosaic, with a variety of habitats varying from sand forest and miombo woodland to open grassland floodplain and swampland. Ungulate populations were once reduced to < 5% of their historic abundance after decades of war but have since displayed dramatic recovery, with post‐war prey biomass currently exceeding 1500 kg/km^2^ across the landscape (Briers‐Louw, Kendon, Rogan, et al. 2024). In contrast, recent studies revealed that leopard, lion ( *Panthera leo* ), and spotted hyaena ( *Crocuta crocuta* ) still occur at relatively low densities (< 3 individuals/100 km^2^; Briers‐Louw, Kendon, Rogan, et al. 2024; Briers‐Louw, Kendon, Ngalijuane, et al. 2024; Briers‐Louw et al. 2025), with cheetah ( *Acinonyx jubatus* ) recently reintroduced (Kendon et al. 2025). Trophy hunting is the primary source of income for these WMAs, with a relatively low number (3.5 ± 0.5) of male leopards harvested annually across the Complex (Briers‐Louw, Kendon, Rogan, et al. 2024).

### 2.2 Leopard Collaring

To capture leopards in this landscape, several methods were initially attempted, but yielded little success due to low detectability and response of leopards. A trained pack of hounds (*n* = 10), a method previously used to safely capture and immobilize a broad range of felids (McBride et al. 2016), was then implemented. This technique yielded higher success, although we emphasize that it should only be applied by trained professionals under the right conditions (Dellinger et al. 2023; Furtado et al. 2008; McBride et al. 2016).

For this method, a 4 × 4 vehicle was used to drive roads at 15 km/h between dawn and dusk in search of fresh leopard tracks. Hounds could not be used during daylight hours due to the presence of tsetse flies and associated diseases (e.g., trypanosomiases). When fresh leopard tracks were detected, hounds were released to follow the leopard scent, with the number of hounds (four to six) dependent on track freshness, terrain, and atmospheric pressure. All hounds were monitored using Garmin GPS collars linked to a central GPS monitor, which indicated the location of all individuals. Trailing distances from the release of hounds to capture ranged from 500 m to 4 km, with < 15 min chase time to the tree. Once a leopard was effectively “treed” by the hounds, the hounds were secured by their handlers to avoid injury and reduce stress on the leopard (McBride et al. 2016). A taut cargo‐capture net and a “bed” of leaves were packed underneath the leopard to protect its fall (Furtado et al. 2008). Leopards were immobilized by a qualified veterinarian using approved techniques and standard drug combinations before being assessed for general health and being fitted with AWT satellite collars (African Wildlife Tracking cc, Pretoria, South Africa), each weighing approximately 650 g (i.e., < 2.2% of leopard body mass and thus within an acceptable range; Swanepoel et al. 2015). One individual was treated and collared after being rescued from a gin trap set by poachers (see Briers‐Louw, Kendon, Rogan, et al. 2024). Leopard ages were estimated based on morphometric indicators (i.e., body weight and measurements) and tooth wear (Stander et al. 1997), while photographs were recorded for identification purposes. Collared leopards were also monitored using a longitudinal camera trapping database to aid identification and cross‐reference age estimation at capture (Balme et al. 2017).

### 2.3 Data Analysis

To investigate leopard spatial ecology, we used continuous‐time movement modeling in the *ctmm* package (Calabrese et al. 2016) in R (R Core Development Team 2023). To determine whether individuals established fixed HRs, we constructed semi‐variograms (i.e., the estimated semi‐variance in relocation data as a function of time lag between locations; Calabrese et al. 2016; Fleming et al. 2014). Range residency was defined by asymptotic smoothing of semi‐variance with increasing time lags (Calabrese et al. 2016). To estimate leopard HRs, we used autocorrelated kernel density estimators (AKDEs), a continuous‐time stochastic process. This method accounts for autocorrelated bivariate Gaussian density estimation for relocation data (Fleming et al. 2014). Two movement models were then performed: (1) Ornstein‐Uhlenbeck (OU) process incorporates a random search model without space‐use limitations (Brownian motion) with a tendency to remain in a finite HR, and (2) Ornstein‐Uhlenbeck Foraging (OUF) process combines velocity autocorrelation time‐scale (i.e., the average time an animal moves in one direction at a constant speed across time lags; a measure of path sinuosity) and limited space use (Calabrese et al. 2016; Fleming et al. 2014; Fleming and Calabrese 2017). Maximum likelihood models were fitted to the data and model ranking was derived from AICc values. Best‐fitting models were used to calculate HRs (95% and 50% isopleths) and movement parameters including HR crossing time, daily distance traveled, and velocity autocorrelation time‐scale for leopards (Calabrese et al. 2016; Fleming and Calabrese 2017). Pairwise HR overlap between female leopards was calculated from AKDE HRs using the Bhattacharyya Coefficient, with values ranging from 0 (no overlap) to 1 (complete overlap). Traditional minimum convex polygon (MCP) analyses and kernel density estimations (KDE) were also conducted using the R package *adehabitatHR* (Calenge 2006) for comparability. Seasonal (i.e., wet and dry) HR sizes were compared using a Wilcoxon signed rank test, with analysis limited to individuals with at least three months of data and a minimum of 70 fixes for each season, which resulted in one to two seasonal pairs per individual.

Observed HR estimates were then contextualized relative to predicted HR sizes based on prey biomass, using the formula (Hayward et al. 2009):

(1) y=−0.564x+3.532

where *y* is the log‐transformed leopard HR size (in km^2^) and *x* is the log‐transformed prey biomass (in kg/km^2^). Prey biomass was derived from the mean estimates between 2019 and 2021 aerial surveys conducted across the Complex (i.e., 1500 kg/km^2^; Briers‐Louw, Kendon, Rogan, et al. 2024).

A camera trapping survey was conducted concurrently in 2021 within the central region of the Complex (619 km^2^), encompassing the ranges of all collared females. Given that no representative telemetry‐based HR estimates were available for male leopards, spatial capture‐recapture (SCR) models were performed in the *secr* package (Efford 2022) to generate sex‐specific parameter estimates for leopards to provide a comparative male HR size, albeit derived from a different method. More detail of the survey design and statistical analysis can be found in Briers‐Louw, Kendon, Rogan, et al. (2024). Both female and male circular HR sizes (95% isopleths) were calculated using the spatial parameter (i.e., sigma, *σ*) derived from the SCR models, with a half‐normal detection function (Efford et al. 2016). All statistical analyses were conducted in R statistical software (R Core Development Team 2023).

## 3 Results

During the study, we collared four adult female leopards, estimated between 2 and 6 years old and weighing 30–45 kg (Table S1). Importantly, no leopards, humans, or hounds were injured during the capture process, and in response, leopards did not display any marked movements away from the capture location. Average monitoring duration of leopards was 16.48 ± 6.72 months (9.63–27.10 months).

All leopards exhibited HR stabilization and range residency as semi‐variance plots reached clear asymptotes, with their movements best characterized by OUF models, based on AICc values (Figure 1; Table 1). Mean leopard HR (95%) and core range (50%) based on the best‐fitting models was 163.78 ± 122.53 (SD) km^2^ and 37.45 ± 29.10 km^2^ respectively (Figure 1, Table 1). The youngest leopard, L3, supported the smallest HR (46.24 km^2^, 95% CI: 31.08–64.37 km^2^), although her movements may have been influenced by the injury sustained from the gin trap and collar malfunction. In contrast, the second youngest leopard, L4, supported the largest HR (364.93 km^2^, 95% CI: 280.38–460.47 km^2^). No significant difference was detected between seasons for HR (*p* = 0.875) or core range sizes (*p* = 0.813). Mean distance traveled by leopards was 7.93 ± 3.22 km/day based on the best‐fitting models (Table S1). Mean HR crossing time for leopards was 4.69 ± 1.87 days and mean velocity autocorrelation timescales were 2.67 ± 1.91 h. HR overlap was relatively high for a single pairwise comparison (L1–L2: 0.60–0.72; Figure S1), although relatively low overall (mean = 0.14 ± 0.11 [SE]; Table S2). Predicted HR size (55 km^2^), based on site‐specific prey biomass, was one‐third of the mean female HR observed in this study (164 km^2^).

**TABLE 1:** Individual details and Auto‐correlated Kernal Density Estimator (AKDE) home‐range (HR; km^2^) estimates of four female leopards collared in the Zambezi Delta, central Mozambique (2019–2023).

Leopard ID	Sex	Monitoring days	Total fixes	Best‐fitting model	Home‐range (km^2^)					
					50% AKDE (95% CI)	95% AKDE (95% CI)	50% MCP	95% MCP	50% KDE	95% KDE
L1	♀	518	1598	OUF anisotropic	27.71 (23.33–32.46)	155.70 (131.08–182.40)	32.22	150.21	21.42	155.79
L2	♀	813	2260	OUF anisotropic	27.50 (24.25–30.95)	88.23 (78.09–98.99)	25.39	56.88	23.93	72.53
L3^a^	♀	289	184	OUF anisotropic	8.58 (5.76–11.94)	46.24 (31.08–64.37)	5.66	22.72	5.80	37.42
L4	♀	358	1944	OUF	86.03 (66.10–108.56)	364.93 (280.38–460.47)	84.26	307.10	66.65	326.47
					37.45 ± 29.10	163.78 ± 122.53	36.88 ± 29.04	134.23 ± 110.18	29.44 ± 22.57	148.05 ± 111.62

*Note:* Minimum Convex Polygon (MCP) and Kernel Density Estimation (KDE) are included for comparisons.

^a^ Female treated and collared after being caught in a poacher's gin trap in August 2021; spatial movements may have been influenced by her injuries and collar malfunction as only 184 fixes were recorded.

The spatial parameter derived from the SCR‐based camera trapping study (Briers‐Louw, Kendon, Rogan, et al. 2024) was used to calculate mean 95% circular HR estimates of 97.8 ± 0.4 (SE) km^2^ for females and 354.7 ± 4.0 (SE) km^2^ for males.

## 4 Discussion

Our findings provide the first robust estimation of leopard HR size in a post‐war context for central Mozambique. The observed leopard HR sizes (46–365 km^2^) fell within the range of global estimates (15–886 km^2^; Snider et al. 2021), yet leopard spatial organization did not entirely conform to established predictions in this mesic environment (i.e., HR size was approximately three times that predicted from prey biomass; Hayward et al. 2009). With complementary evidence indicating that leopard population density is suppressed well below ecological carrying capacity (Briers‐Louw, Kendon, Rogan, et al. 2024), the larger‐than‐expected HR sizes recorded in this study may be a result of low population densities rather than limited resource availability. This study offers unique and valuable insights into the complexity of large carnivore spatial ecology within a post‐war landscape undergoing ecological recovery.

Female leopard HR sizes were most comparable to those recorded in semi‐arid Namibia (104 km^2^, Weise et al. 2015; 188 km^2^, Stander et al. 1997; 52–394 km^2^, Marker and Dickman 2005) and mountainous mixed‐use landscapes in South Africa (164–179 km^2^, Grimbeek 1992; 117–182 km^2^, Müller et al. 2022). Interestingly, HR estimates from a similar high‐rainfall tropical forest in Ivory Coast were substantially lower (25 km^2^), with this population occurring at higher densities (> 7 leopards/100 km^2^; Jenny 1996). Given the relatively low leopard density in our study area, despite abundant prey populations (Briers‐Louw, Kendon, Rogan, et al. 2024), these findings suggest that population density could be a key factor contributing to these larger‐than‐expected HR sizes. The lack of seasonal difference in leopard HR size recorded in this study is likely attributed to the abundance and ubiquity of sedentary prey across the Complex (CEAGRE—Centro de Estudos de Agricultura e Gestão de Recursos Naturais 2024), although other ecological (e.g., year‐round availability of water) and behavioral factors (e.g., aseasonal breeding patterns) could also moderate seasonal HR size variations. Furthermore, the daily distance traveled by female leopards in our study was comparable to that recorded in Namibia (6.8 km/day, Stander et al. 1997), further supporting such space use comparisons. Our study found relatively low average HR overlap, with one instance of high overlap (i.e., L1–L2: 0.60–0.72), with their HRs appearing more spatially adjacent than concentric around a shared resource. This spatial pattern may indicate a mother‐daughter association, although further investigation is required to validate this assumption, especially given the offset monitoring periods for these individuals (Table S1, Figure S2). A study from South Africa found that a relatively high proportion of leopard daughters established HR centroids within their mean maternal HRs (37%–43%) and 1st order mean peripheral HR (30%–43%; Naude et al. 2020), suggesting that such mother‐daughter spatial organization may be reasonably common among leopards. While higher levels of HR overlap are often observed in areas with low leopard density (Marker and Dickman 2005; Périquet et al. 2022), disentangling intra‐sexual overlap remains a challenge due to small sample sizes and the potential for missing neighbors. Based on SCR‐derived sigma values, male HR size was approximately 3.6 times larger than that of females, which broadly aligns with a range‐wide study of inter‐sexual HR differences (i.e., male HRs were 3.2 times larger than females, Snider et al. 2021). However, we apply these estimates with caution as camera traps may have incorporated “transient” individuals in capture histories.

While the relatively small and sex‐specific sample size, coupled with irregular data and sampling periods, poses inherent limitations to this study, the relatively broad age classes of sampled leopards likely offer representative HR variability across this landscape. Additionally, HR sizes and associated parameters were estimated using robust modeling techniques, which correct for such biases in small sample sizes and irregularities (Fleming et al. 2014). Unfortunately, the absence of male telemetry‐based HR estimates does limit intra‐ and inter‐sexual spatial comparability and management‐related inferences, which is particularly important given that males are subject to legal trophy hunting (Briers‐Louw, Kendon, Rogan, et al. 2024). However, the integration of SCR‐based HR estimates (Briers‐Louw, Kendon, Rogan, et al. 2024) in this study offers comparable estimates, which facilitate reasonable inferences, though we recognize inherent biases in deriving such estimates from camera trap surveys. These limitations may include assumptions of circular HRs, trap coverage not encompassing entire HRs, short survey duration, behavioral response to cameras, and sensitivity to detection rates of transients (Efford 2014; Efford et al. 2016).

Our findings coincide with the hypothesis that female leopards, when given the opportunity, may occupy HRs larger than required to satisfy their energetic requirements (Jetz et al. 2004), which we suggest is a result of the low population density (i.e., 1.0–2.8 leopards/100 km^2^; Briers‐Louw, Kendon, Rogan, et al. 2024) and thus reduced intraspecific competition for space. Importantly, Fattebert et al. (2016) found that an over‐harvested leopard population in South Africa exhibited an adaptive strategy of forming matrilineal kin clusters as the socio‐spatial dynamics within the populations were restructured following population recovery over an 11‐year period. In light of this, we predict that, as the Zambezi Delta leopard population recovers, female leopard HRs will likely contract and overlap more as mothers bequeath portions to their philopatric daughters, following the density‐driven and dispersal‐regulated hypothesis (Fattebert et al. 2016; Naude et al. 2020). Although we anticipate that this process may be more gradual in our study area given the low baseline density estimate and threat from anthropogenic pressures across this vast, unfenced landscape. Furthermore, such recovery could also support the restoration of natural ecological processes, facilitating the re‐alignment of natural natal dispersal and outbreeding patterns, thus promoting long‐term population viability (Fattebert et al. 2015). Interestingly, the same South African study also found that male leopards exhibited different spatial patterns compared to females in response to population recovery (Fattebert et al. 2016). As a result, male leopards are expected to maintain relatively large ranges and low overlap among individuals, consistent with dispersal regulated sex‐specific strategies (Fattebert et al. 2016). However, the relatively low but consistent legal harvest of male leopards (Briers‐Louw, Kendon, Rogan, et al. 2024) may influence spatial dynamics both for males (e.g., increased HR size and overlap; Maletzke et al. 2014) and females (e.g., transitioning to resource‐poor habitats; Keehner et al. 2015) as the population recovers and thus requires further investigation.

Large carnivores in central Mozambique were exposed to decades of armed conflict, and along with subsequent anthropogenic pressures, their populations remain suppressed below ecological carrying capacity or have become extirpated (Briers‐Louw, Kendon, Rogan, et al. 2024; Briers‐Louw et al. 2025; Pringle 2017). Bushmeat poaching represents a cryptic and insidious threat to large carnivore populations, either directly through mortality (i.e., bycatch or targeted poaching) or indirectly through prey depletion, with potential negative implications on their socio‐spatial dynamics such as demographic instability and expanded HRs as individual ranges more broadly in search of prey (Almeida et al. 2025; Briers‐Louw, Kendon, Rogan, et al. 2024; Naude et al. 2020). Habitat loss and fragmentation may also force leopards to navigate human‐transformed landscapes, increasing mortality risk associated with conflict and bushmeat poaching. Improved anti‐poaching efforts have dramatically reduced these threats within the central region of the Zambezi Delta, creating an important protected core. However, further enforcement is required along the landscape peripheries to ensure this leopard population recovers and restructures spatially (Briers‐Louw, Kendon, Rogan, et al. 2024), as there may be hidden lag effects on spatial dynamics following anthropogenic perturbation (Fattebert et al. 2016). Furthermore, given that unsustainable male‐biased harvest can result in high male turnover, increased infanticide, and disruption of socio‐spatial stability, trophy hunting quotas and statutory processes that regulate problem animal control should be regularly reviewed and informed by scientific research (Balme et al. 2009; Briers‐Louw, Kendon, Rogan, et al. 2024).

The restructuring of leopard socio‐spatial dynamics in central Mozambique is largely contingent upon the recovery of the resident Zambezi Delta population and the recently reintroduced population in neighboring Gorongosa National Park. Establishing landscape connectivity between these populations will drastically improve the long‐term viability of leopards across this region. Given the mounting evidence of anthropogenic threats suppressing large carnivore populations in Mozambique (Almeida et al. 2025), conservation area management should prioritize efforts by securing existing and potential landscapes (e.g., reducing bushmeat poaching, restricting human encroachment, mitigating human‐wildlife conflict, promoting sustainable trophy hunting, and maintaining prey populations) to provide sufficient safe space necessary for these wide‐ranging carnivores to recover and restructure their complex spatial ecologies. We also strongly recommend continued monitoring of these populations through individual‐based collaring and/or population‐based camera trapping to assess longitudinal shifts in population‐level demographics and socio‐spatial dynamics of leopards and other large carnivores in Mozambique.

## Author Contributions

**Willem D. Briers‐Louw:** conceptualization (lead), data curation (lead), formal analysis (lead), investigation (lead), methodology (lead), project administration (lead), visualization (lead), writing – original draft (lead), writing – review and editing (lead). **Tamar Kendon:** writing – review and editing (supporting). **Andres Hayes:** writing – review and editing (supporting). **David Gaynor:** conceptualization (supporting). **Vincent Naude:** writing – original draft (equal), writing – review and editing (equal).

## Ethics Statement

This research was conducted under a research permit (ANAC RP# 06‐10‐2023) from the National Administration of Conservation Areas (ANAC) in Mozambique. Ethical approval for this study was obtained from the Institutional Animal Care and Use Committee (IACUC #23‐010), and permission was obtained from the concessionaire of Coutada 11 to conduct research in this wildlife management area.

## Conflicts of Interest

The authors declare no conflicts of interest.

## Supporting information


**Appendix S1:** ece372312‐sup‐0001‐AppendixS1.docx.

## Data Availability

All relevant data are within the manuscript, Supporting Information S1, and on figshare (https://doi.org/10.6084/m9.figshare.29974849; https://doi.org/10.6084/m9.figshare.29974909).
