# Supplementary material for: Stretching Out: Leopard Home‐Range Expansion in Response to Suppressed Population Density in a Recovering Post‐War Landscape
Source: Ecol Evol. 2025 Oct 10;15(10):e72312. doi: 10.1002/ece3.72312 (PMC12513734; doi:10.1002/ece3.72312)
Supplement: Supplementary file 1 — Appendix S1: ece372312‐sup‐0001‐AppendixS1.docx. [file ECE3-15-e72312-s001.docx]

# **Stretching out: leopard home-range expansion in response to suppressed population density in a recovering post-war landscape**

**Appendix S1.** Code of home range analyses. The code that support the findings of this study are openly found in fighare (DOI: 10.6084/m9.figshare.29974909).

**Appendix S2.** Leopard collar dataset. The data that support the findings of this study are openly found in fighare (DOI: 10.6084/m9.figshare.29974849).

**Table S1.** Individual leopard details and home range parameter estimates of four female leopards in the Zambezi Delta, Mozambique between 2019 and 2023.

| ID | Sex | Estimated age  (years) | Weight (kg) | Start date | End date | Home range crossing time (days) | Velocity auto-correlation timescale (hours) | Daily distance travelled (km) |
| --- | --- | --- | --- | --- | --- | --- | --- | --- |
| L1 | ﻿♀ | 5–6 | 45 | 01-Oct-2019 | 02-Mar-2021 | 3.37  (2.78–4.08) | 1.64  (1.34–2.00) | 8.24  (7.56–8.94) |
| L2 | ﻿♀ | 4–5 | 43.5 | 24-Jul-2021 | 15-Oct-2023 | 2.42  (2.21–2.77) | 0. 79  (0.72–0.87) | 10.64  (10.24–11.05) |
| L3 | ﻿♀ | 2–3 | 30 | 09-Aug-2021 | 25-May-2022 | 7.44  (4.60–12.02) | 3.04  (1.54–5.99) | 2.31  (1.76–2.89) |
| L4 | ﻿♀ | 2–3 | 33 | 01-Nov-2019 | 24-Oct-2020 | 5.33  (4.06–7.00) | 1.62  (1.48–1.78) | 9.34  (9.01–9.68) |
|  | |  |  |  |  | 4.64 ± 1.93 | 2.10 ± 0.66 | 7.63 ± 3.19 |

**Table S2.** Pairwise home range overlap, based on the Bhattacharyya Coefficient, for female leopards in the Zambezi Delta of central Mozambique. Values in brackets indicate 95% confidence intervals (CIs).

| ID | L1 | L2 | L3 | L4 |
| --- | --- | --- | --- | --- |
| L1 |  | 0.66  (0.60-0.72) | 0.12  (0.08-0.17) | 0 |
| L2 | 0.66  (0.60-0.72) |  | 0 | 0 |
| L3 | 0.12  (0.08-0.17) | 0 |  | 0.07  (0.03-0.15) |
| L4 | 0 | 0 | 0.07  (0.03-0.15) |  |


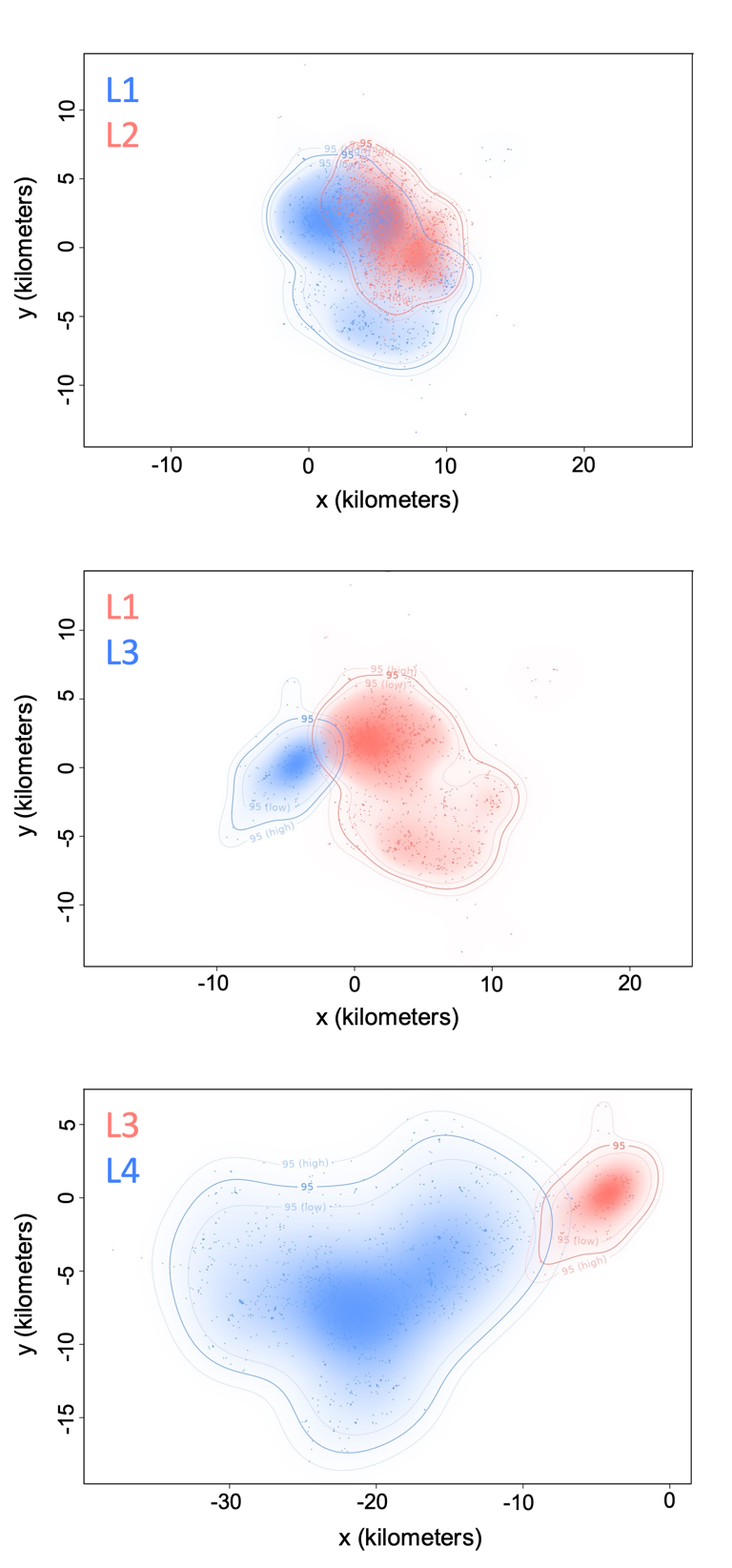
**Figure S1.** Pairwise home range overlap, based on the Bhattacharyya Coefficient, for female leopards in the Zambezi Delta of central Mozambique. Dark lines indicate estimated home range size and lighter lines indicate 95% confidence intervals (CIs). The heatmap represents areas of more intensive spatio-temporal utilisation density, while the dots indicate fixes for each individual.

**
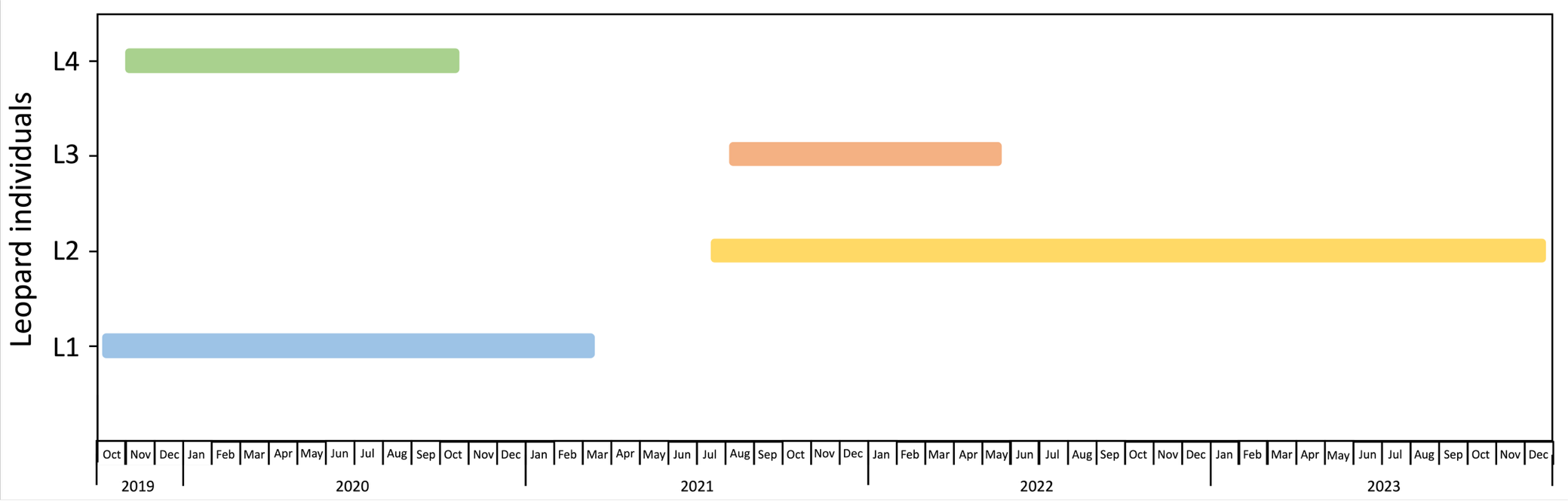
Figure S2.** Diagram illustrating the monitoring timelines for all leopards in this study.
